# Serpin Treatment Suppresses Inflammatory Vascular Lesions in Temporal Artery Implants (TAI) from Patients with Giant Cell Arteritis

**DOI:** 10.1371/journal.pone.0115482

**Published:** 2015-02-06

**Authors:** Hao Chen, Donghang Zheng, Sriram Ambadapadi, Jennifer Davids, Sally Ryden, Hazem Samy, Mee Bartee, Eric Sobel, Erbin Dai, Liying Liu, Colin Macaulay, Anthony Yachnis, Cornelia Weyand, Robert Thoburn, Alexandra Lucas

**Affiliations:** 1 Department of Medicine, University of Florida, Gainesville, Florida, United States of America; 2 Department of Molecular Genetics & Microbiology, University of Florida, Gainesville, Florida, United States of America; 3 Department of Ophthalmology, University of Florida, Gainesville, Florida, United States of America; 4 Department of Pathology, University of Florida, Gainesville, Florida, United States of America; 5 Viron Therapeutics, London, Ontario, Canada; 6 Division of Immunology and Rheumatology, Stanford University School of Medicine, Stanford, California, United States of America; INSERM-Université Paris-Sud, FRANCE

## Abstract

Giant cell arteritis (GCA) and Takayasu’s disease are inflammatory vasculitic syndromes (IVS) causing sudden blindness and widespread arterial obstruction and aneurysm formation. Glucocorticoids and aspirin are mainstays of treatment, predominantly targeting T cells. Serp-1, a Myxomavirus-derived serpin, blocks macrophage and T cells in a wide range of animal models. Serp-1 also reduced markers of myocardial injury in a Phase IIa clinical trial for unstable coronary disease. In recent work, we detected improved survival and decreased arterial inflammation in a mouse Herpesvirus model of IVS. Here we examine Serp-1 treatment of human temporal artery (TA) biopsies from patients with suspected TA GCA arteritis after implant (TAI) into the aorta of immunodeficient SCID (severe combined immunodeficiency) mice. TAI positive for arteritis (GCA^pos^) had significantly increased inflammation and plaque when compared to negative TAI (GCA^neg^). Serp-1 significantly reduced intimal inflammation and CD11b^+^ cell infiltrates in TAI, with reduced splenocyte Th1, Th17, and Treg. Splenocytes from mice with GCA^pos^ grafts had increased gene expression for interleukin-1beta (IL-1β), IL-17, and CD25 and decreased Factor II. Serp-1 decreased IL-1β expression. In conclusion, GCA^pos^ TAI xenografts in mice provide a viable disease model and have increased intimal inflammation as expected and Serp-1 significantly reduces vascular inflammatory lesions with reduced IL-1β.

## Introduction

Inflammatory vasculitic syndromes (IVS) produce widespread activation and invasion of innate immune cells throughout all layers of medium to large arterial branches [1]. Giant cell arteritis (GCA) occurs predominantly in patients 50 years or older and Takayasu’s disease, also called pulseless disease, is detected in younger patients. Both are termed IVS [2]. GCA and Takayasu’s disease are perceived as different spectrums of similar IVS disorders, both causing progressive disease, with variable presentations of cerebral ischemia and jaw claudication, sudden loss of vision, peripheral gangrene, organ ischemia, thoracic aneurysms and cardiomyopathy [1,2]. Polymyalgia rheumatica increases GCA risk with an estimated prevalence of 200 per 100,000 [3]. Sudden blindness occurs in GCA when the posterior ciliary artery is occluded and is considered a medical emergency. Risk for sudden blindness is diagnosed by temporal artery (TA) biopsy with urgent initiation of steroid therapy. Steroids and aspirin [4] are the mainstays of treatment for GCA. In a double-blind, placebo controlled study employing glucocorticoid pulse therapy, patients treated at diagnosis had less recurrence. However, while glucocorticoid treatment is effective, responses may be delayed and inflammatory infiltrates can persist [1–4]. Aspirin treatment is also associated with improved outcomes potentially through combined inhibition of inflammation and platelet activation. However, disease can persist and loss of vision can occur despite immediate treatment. Additionally, recurrence can occur after steroid withdrawal [4–6]. Attempts to introduce steroid-sparing agents, such as inhibitors of tumor necrosis factor alpha (TNFα) and methotrexate have been less successful [5,7,8]. TNFα inhibition was ineffective while methotrexate and therapies targeting IL-6 had variable benefit. Severe Takayasu’s disease remains even more difficult to treat.

In a recent study in a mouse model of IVS using a lethal Mouse Herpesvirus 68 (MHV68) infection-induced vasculitis, Serp-1 markedly reduced arterial and systemic inflammation and improved survival [9]. Serp-1 is a 55 kDa myxomaviral serpin, inhibiting inflammatory responses in animal models of vascular injury and organ transplant [10–13], via blockade of thrombolytic proteases, tissue- and urokinase- type plasminogen activators (tPA and uPA), as well as plasmin and thrombotic protease factor X (fX) [10,12] and factor II (fII, thrombin). Serp-1 modifies macrophage, and T helper (Th) responses in mouse aortic transplants [12,13] and macrophage responses in MHV68-induced vasculitis [9].

In atherosclerotic coronary artery disease, serine proteases in the coagulation and fibrinolytic pathways contribute to unstable angina and sudden vascular occlusion [13]. Inflammatory cell responses activated by serine protease cascades are regulated by *ser*ine *p*rotease *in*hibitors, termed *serpins*. Both thrombosis as well as inflammatory mononuclear cell invasion are seen in GCA lesions. Granulomatous inflammatory infiltrates composed of macrophages, CD4^+^ T cells, and multinucleated giant cells are pathognomonic of GCA and are associated with intimal hyperplasia causing luminal compromise [14]. Dendritic cells (DCs) act as sentinels and signal T-cell infiltration into the vessels on encountering pathogen-derived motifs. Aberrations in this signaling have been shown to be the cause of T-cell hyper-responsiveness and are implicated in GCA [14–16]. Two separate pathogenic pathways mediated by Th1 and Th17 cells, respectively contribute to GCA and steroid treatment has greater effects on Th17 and not Th1 [6]. Thus, current therapy may not address all inflammatory responses that drive IVS.

The rationale and aims for performing these studies is as follows. Our first aim was to examine the capacity to test for anti-inflammatory activity and disease progression using human specimens, in this case temporal artery biopsy specimens, embedded in a mouse aorta. Our second aim was to assess the capacity of the myxomavirus-derived serpin, Serp-1, to modify disease progression in the inflammatory vasculitic syndromes (IVS) such as GCA or Takayasu’s disease, using human TA specimens embedded in a mouse aorta. In order to examine the potential for Serp-1 treatment to reduce inflammation in GCA, we have established an animal model of GCA, modified from the Weyand model of TA subcutaneous implant [15–17]. In this modified model, human TA biopsy specimens from patients with suspected GCA were inserted as full thickness grafts directly into the anterior wall of the abdominal aorta in SCID mice, termed a TAI xenograft, providing direct contact with circulating blood flow to more closely approximate normal physiologic conditions. Vascular inflammatory lesion (VIL) growth and inflammatory cell responses in GCA^pos^ and GCA^neg^ TAI grafts were measured with and without Serp-1 treatments.

## Methods

### Human Temporal Artery Biopsy and Peripheral Blood Mononuclear Cell (PBMC) Isolates

All procedures concerning human TA biopsy and PBMC isolates were approved by the University of Florida Institutional Review Board (IRB) and conform to national standards. All patients gave written informed consent and only patients undergoing clinically indicated TA biopsy performed by a surgeon for suspected GCA were enrolled. TA biopsies were independently identified as GCA^pos^ (3 biopsies) or GCA^neg^ (7 biopsies) by standard pathologic indices. TA specimens were stored frozen at -80^°^C in OCT medium with no fixative (Sakura Finetek USA, Inc, Torrance, CA, USA) prior to implant. CellTiter 96 Non-Radioactive kit (Promega Corp., Madison, WI, USA) was used with the minced tissue to measure the cell viability of the frozen arterial sections. Once the model was established the survival rate was high (~100%) and this high survival for animals with GCA^neg^ and GCA^pos^ TA transplants suggests that tissue integrity was preserved. PBMCs were isolated from a normal volunteer, collected and frozen at different times, and were pooled and enriched by Ficoll–Paque and followed by centrifugation (GE Healthcare Biosciences, PA, USA). Cell viability was evaluated by Trypan blue exclusion.

### Mouse TAI Xenografts

All animal protocols were approved by University of Florida Institutional Animal Care and Use Committee (IACUC) and conform to national guidelines. All animals received care in compliance with the Principles of Laboratory Animal Care and National standards.

Ten TA biopsy specimens from patients with suspected GCA were implanted into 32 NOD.CB17-*Prkdc* ^*SCID*^/J recipient mice (Jackson Lab, Sacramento, CA, USA) 20–25 gm, 6–8 weeks of age. Each biopsy specimen was divided into 2 to 4 sections for TAI into SCID mice, with either Saline (100 μl; N = 10 mice), Serp-1 (100 ng/g/100 μl; N = 10), PBMC + Saline (5×10^6^ PBMC cells / 100 μl saline; N = 6), or PBMC + Serp-1 (5×10^6^ PBMC cells + 100 ng/g/100 μl; N = 6) treatment (Table 1). Saline or Serp-1 treatment was given immediately after surgery by intravenous (IV) injection with subsequent daily intraperitoneal (IP) injections for 9 days. PBMC infusions were given as a one-time IV bolus post-operatively.

**Table 1:** Temporal artery implant (TAI) numbers and treatment.

Donor	Recipient	TAI numbers	Treatment	Follow up	Survival
TAI	SCID	10	Saline	28 days	10/10
TAI	SCID	6	PBMC + Saline	28 days	5/6
TAI	SCID	10	Serp-1	28 days	10/10
TAI	SCID	6	PBMC + Serp-1	28 days	6/6

PBMC (5×10^6^ / 100 μl saline); Serp-1(100 ng/g / 100 μl saline); Saline (100 μl)

**Fig 1:**
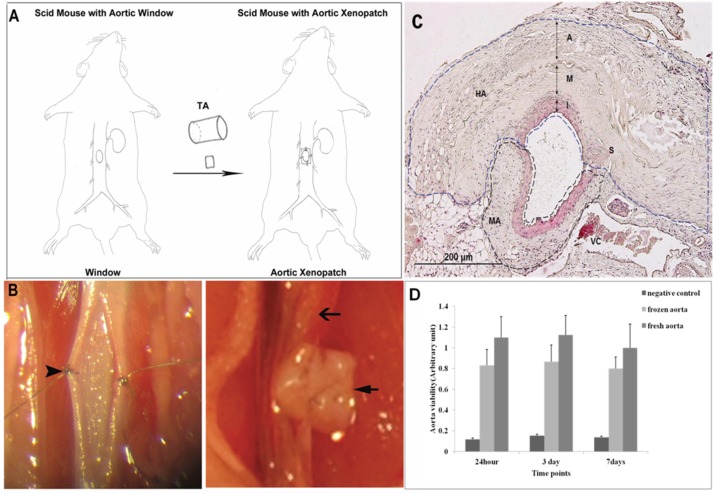
Aortic Window Xenograft / TAI Model. (A) Schematic of human temporal artery biopsy implant into the aorta of SCID mice. (B) Left panel—surgical window in the anterior abdominal aortic wall, Right panel—TA xenopatch covering the surgical window. Arrowhead—surgical window in recipient mouse, Short black arrows—donor human TAI graft. Longer black arrows—recipient mouse aorta. (C) Cross section of TAI biopsy graft in SCID mouse aorta. Blue line outlines TA graft (HA—human artery). Black line outlines mouse artery (MA). (D) Mouse aortic cells display excellent viability after storage in OCT at -80°C for 1–7 days. I—intima). M—media, A—adventitial, VC—vena cava, S—suture.

This xenograft model is adapted from the subcutaneous implant model (Stanford University, CA), in order to provide a more natural physiological interaction between the TAI graft and circulating blood cells [2,4,14,18]. For TAI xenografts, the aorta of SCID mice, anesthetized with ketamine/xylazine given IP, was exposed under sterile conditions and clamped below the renal arteries as previously described for aortic allograft transplantation [10,12]. An ~ 0.1×0.3 cm^2^ window was surgically opened on the abdominal aorta between vessel clips and a human TA biopsy xenograft of similar size implanted (Fig. 1A-C) using 11–0 monofilament nylon sutures (ARO Surgical, Newport Beach. CA, USA). Vessel clips were then removed and medications given once vessel pulsation restored. Of two SCID mice that received sections from the same TA biopsy specimen; one was given saline and the other Serp-1. When PBMC infusion was given, sections from the same TA biopsy were implanted into two additional SCID mice, one of which received saline with PBMC and the other Serp-1 with PBMC in addition to the two mice receiving TAI with either Saline or Serp-1 treatment without PBMCs. PBMC donor-matched to the TA biopsies were not available. One TA engrafted mouse with PBMC infusion died at 27 days with thrombosis. There were no other early losses after TAI surgery. At four weeks follow-up, mice were euthanized by IP injection (Euthanyl, Virbac AH Inc., TX, USA). Aortas and spleens were collected from the mice and analyzed as described below.

### Histological and Morphometric Analysis

Arterial sections were fixed in neutral buffered formalin, embedded, cut into 4 μm cross sections, and stained with haematoxylin and eosin (H & E), as previously described [10,12]. VIL thickness, area, and invading mononuclear cell counts in the medial layers in sections containing TAI grafts were measured by morphometric analysis using an Olympus DP71 camera attached to an BX51 microscope (Olympus America Inc., Center Valley, PA) and quantified using Image Pro 6.0 (MediaCybernetics Inc., Bethesda, MD) [10,12]. For morphometric analysis each aortic specimen was divided into three sections and two cross-sections from each of these three (0.4 μm in width) were stained. The TAI was identified by the sutures placed at the time of implant surgery. The site with the largest intimal thickness section was measured for all specimens. The mean intimal thickness was calculated for each animal and the values were normalized to their corresponding medial thicknesses to obtain the intimal/medial thickness ratios.

Immunohistochemical staining was performed using a HRP/DAB Detection IHC kit (Abcam, Cambridge, MA, USA) and counterstained with haematoxylin [10,12,13]. Sections were stained with Human specific antibodies: Mouse monoclonal to Mitochondria 1:400 ab92824, rabbit monoclonal anti-MCH class 1 1:250, ab52922; rabbit anti-human CD3 1:100 ab93077 and antibodies with cross reactivity to human and mouse: rabbit polyclonal CD3 1:100 ab5690; rabbit polyclonal CD11b 1:400 ab75476; rabbit polyclonal to CD83 1:100 ab64875; rabbit polyclonal to CCR6 1:500 ab78429 (Abcam, Cambridge, MA, USA). Positively stained inflammatory cells in each TAI were counted at sites with the largest numbers of invasive cells and the means calculated for each test animal in three high power field areas (100X oil immersion) in each arterial layer for each engrafted artery.

### Flow cytometry

Mouse splenocytes were isolated as previously described [12] with a final concentration of 5×10^6^ cells/mL. S1 Table lists the immune cell types analyzed and corresponding fluorochrome-labeled antibodies. Cells were mixed with permeabilization buffer (eBioscience, San Diego, CA, USA), centrifuged, resuspended and analyzed by flow cytometry as previously described [12] using a CyAn ADP Analyzer (Dako, Ft Collins, CO, USA). Flow data was analyzed using Gatelogic software (eBioscience).

### RT-PCR Array

Spleens were collected in RNA later (Ambion, Austin, TX, USA) and RNA isolated using RNeasy Mini kit following the manufacturer’s protocol (QIAGEN, Valencia, CA, USA). RNA (500 ng each) was reverse transcribed to cDNA using Superscript VILO cDNA Synthesis kit (Invitrogen Corporation, 11754–250, California, USA) and Real Time PCR was performed using SYBR Green Core Reagent kit and a 7300 RT-PCR system (Applied Biosystems, Austin, TX, USA). Primers are listed in S2 Table. GAPDH was used for normalization.

### Serpin Expression and Purification

Serp-1 was provided by Viron Therapeutics, Inc. (London, ON, Canada). In brief, Serp-1 is purified from the supernatant of recombinant Chinese hamster ovary (CHO) cells by sequential column chromatographic separation with greater than 95% purity as determined by overloaded Coomassie-stained SDS-PAGE gels and reverse-phase HPLC [10,12,19]. Clinical grade, endotoxin free Serp-1 was used for all experiments.

### Statistical Analysis

Statistical analysis was performed using Statview V5.01 (Cary, NC, USA). Mean VIL and medial thickness and cell count from three sections per xenograft were analyzed by analysis of variance (ANOVA) with Fishers PLSD (Protected Least Significant Difference) and unpaired two tailed Student’s T-test secondary analysis (P ≤ 0.05 considered significant).

## Results

### TAI—Aortic Window Xenopatch Model

**Fig 2:**
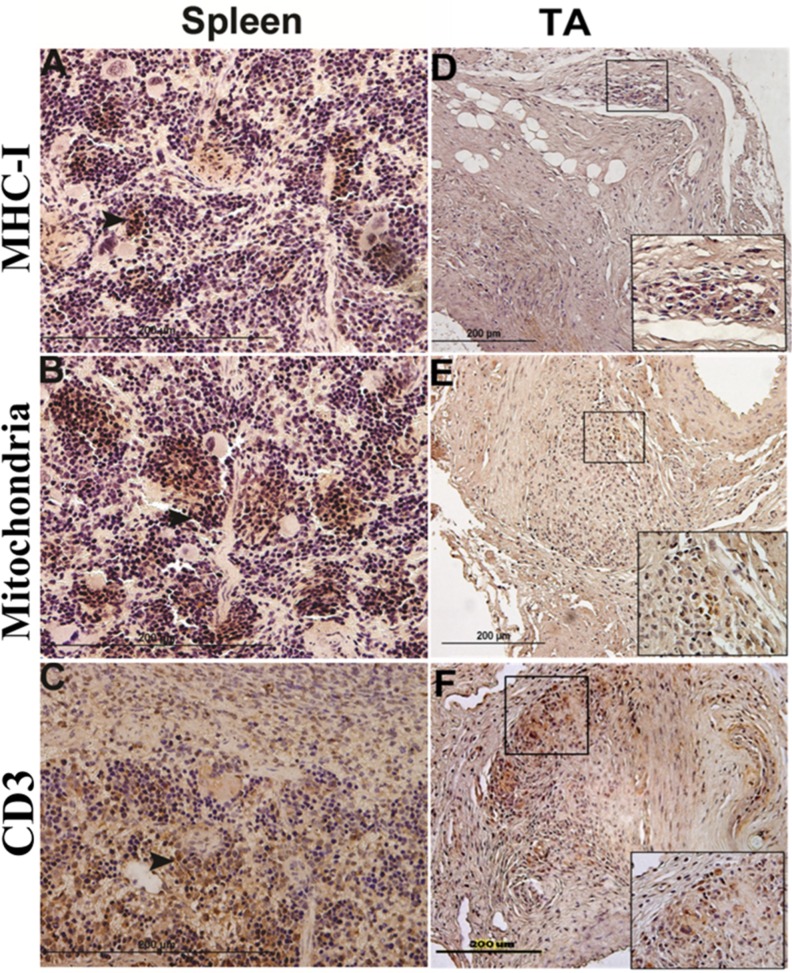
Immunostained human cells in spleen and temporal artery (TA) xenograft sections demonstrate persistent human PBMC colonization at 28 days. (A) MHC-1 positive staining. (B) Mitochondria positive staining. (C) CD3^+^ positive staining. Brown stained cells (arrow) are positively stained for selected human antigens (n = 11. MHC-I—major histocompatibility complex-I-I. Magnification: 400X for spleen, 200X for TA grafts and 800X for insets.

Excellent cell viability was detected in aortic specimens stored at -80°C in OCT (Fig. 1D). Aortic sections were assessed for altered VIL growth and inflammatory cell invasion in areas with GCA^pos^ and GCA^neg^ TAI grafts. In a subset of mice, unmatched PBMC from healthy donors were infused in order to assess the effects of circulating mononuclear cells on arterial inflammatory lesion progression as SCID mice lack lymphocytes. At 4 weeks, survival rate in TAI engrafted mice was 96.9% (Table 1). When invading human PBMC were traced by immunostaining, cells in both spleen (Fig. 2A-C) and xenograft adventitia (Fig. 2D-F) stained positively for human MHC-І (Fig. 2A,D), mitochondria (Fig. 2B,E), and CD3^+^ (Fig. 2C,F).

In summary, a viable TAI xenograft model was established in SCID mice designed to assess inflammation and VIL growth (inflammatory intimal hyperplasia) providing a method to analyze IVS progression in TA biopsy specimens implanted into the aorta of SCID mice with exposure to circulating blood flow.

### Vascular Inflammatory Lesion (VIL) Growth in TAI Xenografts

**Fig 3:**
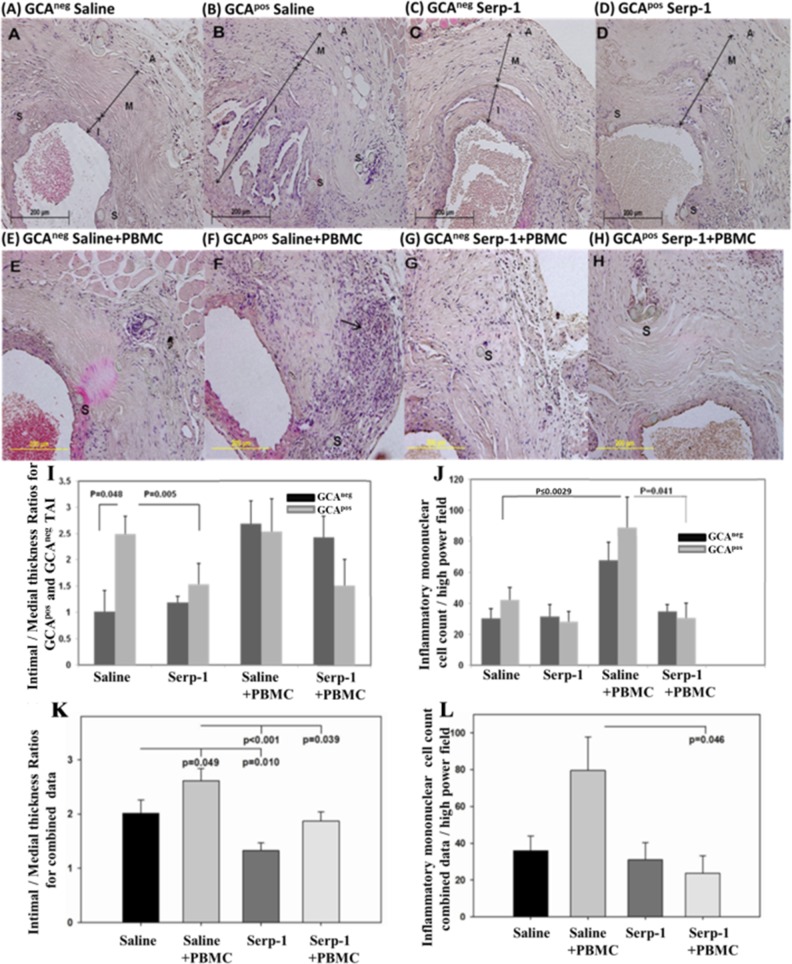
GCA^pos^ TAI grafts have increased vascular inflammatory lesion (VIL) thickness when compared to GCA^neg^ TAI; Serpin treatment reduces intimal thickness. H & E stained cross sections of TAI engrafted SCID mouse aorta at 4 weeks follow up; (A) GCA^neg^ TAI graft saline treated, (B) GCA^pos^ TAI saline treated, (C) GCA^neg^ section, Serp-1 treated, (D) GCA^pos^ section, Serp-1 treated. Invading mononuclear cells in TAI engrafted aortic cross sections in mice after PBMC infusion at 4 weeks: (E) GCA^neg^ cross section, Saline treated, (F) GCA^pos^ section, Saline treated, (G) GCA^neg^, Serp-1 treated. (H) GCA^pos^ Serp-1 treated. (I) Bar graphs of mean intimal/ medial (I/M) thickness ratios (± SE) for TAI grafts. GCA^pos^ TAI with Saline treatment have increased VIL. I/M are significantly reduced with Serp-1 (p = 0.005). (J) Inflammatory cell counts are significantly reduced with Serp-1 treatment in GCA^pos^ implants after PBMC transfusion (p ≤ 0.041; n = 3 each for GCA^neg^ and ^pos^). (K) When the GCA^pos^ and GCA^neg^ data are combined, mean I/M thickness ratios are significantly reduced in Serp-1 treated mice when compared to Saline and PBMC/Saline groups (P ≤ 0.011 and 0.039, respectively; n = 5–10). (L) The mean mononuclear cell counts in combined GCA^pos^ and GCA^neg^ data was significantly reduced in mice treated with Serp-1 after PBMC transfusion but not without PBMC, when compared to controls (p ≤ 0.046; n = 5–10). (I—intima, M—media, A—adventitia, S—suture. Arrow—inflammatory cell. Mag-100–200X).

Intimal VIL were compared in GCA^pos^ and GCA^neg^ xenografts. TAI xenografts (Fig. 3) were identified by sutures around each implant, TAI location on anterior abdominal aortic wall and greater thickness of human TA biopsy sections when compared to mouse aorta. Intimal (I) and medial (M) thickness of TAI was measured and the I/M ratios calculated to normalize for potential differing TA implant sizes (Fig. 3).

In saline-treated animals, without PBMC infusion, the ratio of intimal to medial thickness was increased in GCA^pos^ TAI (Fig. 3B,I) when compared to GCA^neg^ TAI grafts (Fig. 3A,I, P ≤ 0.048). With PBMC infusion, the increase in intimal to medial thickness ratios in GCA^pos^ (Fig. 3I) was no longer significant, on comparison to GCA^neg^ grafts (Fig. 3I). In TAI with no PBMC infusion, no difference was observed for invading mononuclear cells in the medial layer of GCA^pos^ and GCA^neg^ grafts (Fig. 3J). With PBMC infusion, GCA^pos^ implants had significantly increased numbers of invading cells (Fig. 3F,J) when compared to GCA^pos^ sections without PBMC.

### Serp-1 Treatment Reduced Inflammatory TAI Xenograft Vascular Lesions

Serp-1 significantly reduced intimal/medial thickness ratios in GCA^pos^ specimens (Fig. 3D,I) without PBMC infusion, when compared to saline treatment (Fig. 3B,I; P = 0.005). In GCA^neg^ engrafted mice, Serp-1 treatment (Fig. 3C,I) had minimal effect on VIL in mice with or without PBMC infusion. In GCA^pos^ engrafted mice with PBMC infusion, Serp-1 treatment demonstrated a trend toward reduced TA intimal/medial thickness ratios (Fig. 3,I; P = 0.089). Serp-1 significantly reduced the inflammatory cell counts in the medial layer after PBMC transfusion in GCA^pos^ specimens (p ≤ 0.041, Fig. 3 H,J).

As the numbers of implanted TAI were relatively small and IVS lesions can be present as ‘skip lesions’, varying from arterial section to section, and as all the sections were collected from patients with suspected GCA, we assessed whether significant changes were detected when combining data for GCA^pos^ and GCA^neg^ implants. When data from both GCA^pos^ and GCA^neg^ were combined, the intimal to medial thickness ratios were again reduced for Serp-1 treatment (Fig. 3K, P = 0.010). Invading cell counts in the medial layer with PMBC infusion were again increased significantly (P = 0.049). PBMC transfusion induced a marked increase in intimal/medial thickness in saline treated mice, (P = 0.049). A significant decrease in intimal/medial thickness was detected after Serp-1 treatment, with or without PBMC transfusions on analysis of combined data for GCA^pos^ and GCA^neg^ grafts (Fig. 3K; P ≤ 0.001 and P ≤ 0.039). Inflammatory cell counts in the medial layer were reduced with Serp-1 after PBMC transfusion in combined GCA^pos^ and GCA^neg^ grafts (Fig. 3,L P ≤ 0.046).

### Serp-1 Treatment Altered Inflammatory Responses after PBMC Infusion

Peripheral blood mononuclear cell (PBMC) isolates were infused immediately after TAI surgery in order to potentially assess effects of circulating human leukocytes on human TAI graft inflammation and VIL growth/ thickness. SCID mice have markedly reduced lymphocyte counts. PBMC isolates contain neutrophil, lymphocytes (T, B and NK cells), DC, monocytes, and basophils. To explore the inflammatory cells in TAI grafts and cells targeted by Serp-1, aortic sections were stained for human CD3, CD11b, CCR6, and CD83 antibodies, to roughly identify T cell, macrophage, T memory cell, and DCs, respectively. These cell types have been reported to modify TA biopsy inflammation after subcutaneous implantation in SCID mice.

**Fig 4:**
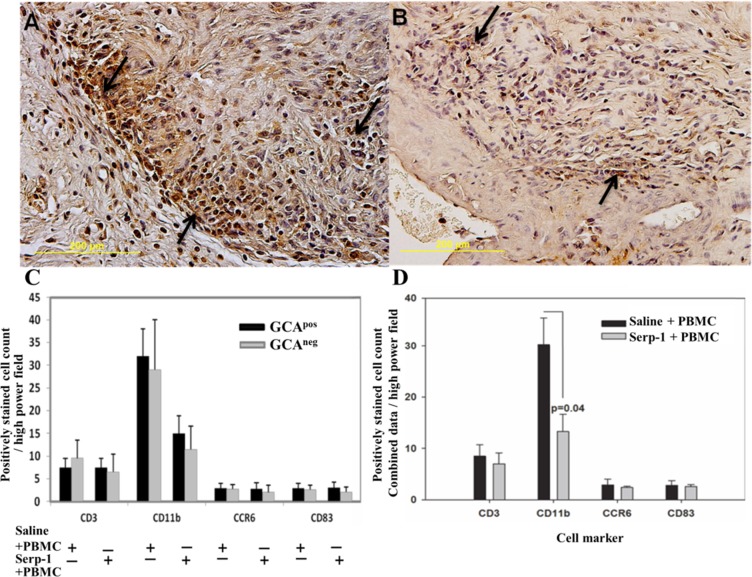
Immunohistochemical analysis demonstrates significantly reduced CD11b positive cell counts with Serp-1 treatment in TAI grafts in SCID mice at 4 weeks (n = 5–10). (A) CD11b^+^ staining of GCA^pos^ sections in Saline + PBMC treated mice. Brown stained areas are positively stained cells (marked by arrows) (B) CD11b^+^ staining of GCA^pos^ sections in TAI grafts with Serp-1 + PBMC treatment. (C) GCA^pos^ or GCA^neg^ engrafted sections without PBMC did not show a significant change for CD3, CD11b, CCR6 nor CD83 stained cell counts (n = 3 each for GCA^pos^ or GCA^neg^). (D) CD11b^+^ cells were significantly reduced by Serp-1 treatment after PBMC transfusion when combining data for GCA^pos^ and GCA^neg^. No difference in CD3^+^, CCR6^+^, and CD83^+^ cell counts were detected for combined data (n = 5–10). Magnification- 400X.

TAI grafts from GCA^pos^ and GCA^neg^ TA biopsy implants displayed minimal if any difference between inflammatory cell types. Serp-1+PBMC treatment in GCA^pos^ and GCA^neg^ specimens produced a trend toward a reduction in CD11b^+^ cells in sections whether GCA^pos^ or GCA^neg^, compared to Saline+PMBC, but did not reach significance (Fig. 4A, 4B and 4C). After PBMC infusion when assessing a combined analysis of all sections, whether GCA^pos^ or GCA^neg^, Serp-1 significantly reduced CD11b^+^ cells in engrafted arteries (Fig. 4D; P ≤ 0.04). No differences in CD3^+^, CCR6^+^, and CD83^+^ cells were detected with Serp-1 treatment (Fig. 4D).

In summary, human CD11b^+^ cell counts in TAI grafts were significantly reduced with Serp-1 treatment when analyzing data from all GCA^pos^ and GCA^neg^ TAI with PBMC.

### Serp-1 Treatment Altered Inflammatory Cell Populations in Spleen Cell Isolates from TA-SCID Chimeras

**Fig 5:**
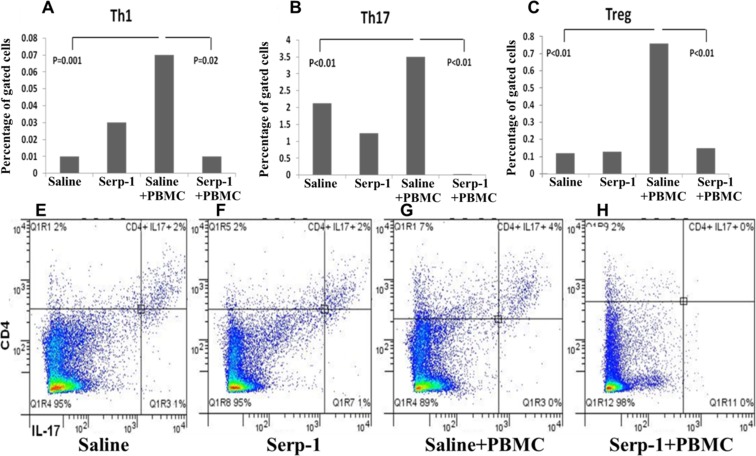
Flow cytometry illustrates decreased inflammatory cell numbers after Serp-1 treatment in splenocytes from TAI engrafted SCID mice. A, B, C and D are representative flow cytometry results for CD4+IL-17+ cells treated by Saline, Serp-1, Saline+PBMC and Serp-1+PBMC respectively. E, F and G show the increased Th1 (CD4+ IFN-γ+), Th17 (CD4+IL-17+) and Treg (CD4+FoxP3+) cells in PBMC transfused TAI engrafted SCID mice when compared to Saline treated mice (P ≤ 0.01), respectively. Serp-1 with PBMC transfusion decreased the cell numbers when compared to PBMC transfusion alone (P ≤ 0.02, P ≤ 0.01, P ≤ 0.01). For Th1 cells, the absolute positive cell count was low, but still a trend can be noted. To increase cell numbers, spleen cells were pooled from the same group.

Systemic immune cell response after TAI engrafting was assessed by flow cytometry of spleen cell isolates (Fig. 5). Cytotoxic T cells, Th1, Th2, Th17, and Treg cells, B cells, hematopoietic stem cells, NK cells, monocytes, DCs (immature and mature), and memory T cells were assessed. Th1, Th17 and Treg cell (Fig. 5 A,B and C), counts were increased by 1.5 fold or greater in PBMC transfused TAI engrafted mice when compared to saline treated mice with no PBMC (p ≤ 0.01). Serp-1 treatment given concurrently with PBMC transfusion decreased Th1 (P < 0.02), Th17 (P ≤ 0.01), and Treg (P ≤ 0.01) cells greater than 3 fold when compared to PBMC / Saline transfusion. Spleen cell counts were low necessitating pooling of cells from the same treatment groups (1.5–3×10^5^). Only changes detected in Th1, Th17, and Treg cells were considered significant and only flow sorting numbers for these cells are reported (Fig. 5).

### Serp-1 Treatment Alters Coagulation and Immune Cell Marker Expression in Spleens

**Fig 6:**
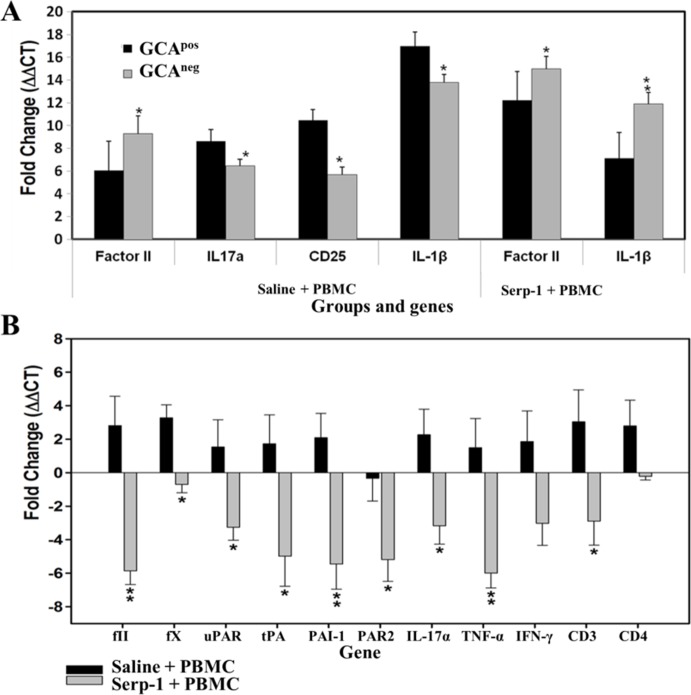
Gene expression changes in splenocytes isolated from mice with TAI after Saline and Serp-1 treatments. (A) Real-time PCR analysis demonstrates significantly increased gene expressions for IL-1β, IL-17, and CD25, in spleen isolates from Saline+PBMC treated GCA^pos^ TA implanted mice (P ≤ 0.0317, 0.0281, and 0.0354, respectively). fII and IL-1β gene expression in GCA^pos^ engrafted mice are significantly reduced by Serp-1+ PBMC treatment (P ≤ 0.002; n = 3 for GCA^pos^ and GCA^neg^) (B) In combined data of GCA^pos^ and GCA^neg^ engrafted mice, PCR array analyses demonstrate significantly increased expression of thrombotic and thrombolytic proteases, serpins and receptors, inflammatory cell markers and cytokines/chemokines in TAI engrafted mice transfused with PBMCs. Serp-1+PBMC treatment significantly reduced expression when compared to Saline + PBMC. (* P ≤ 0.05, ** P ≤ 0.01; N = 4 for Saline + PBMC, N = 5 for Serp-1 + PBMC).

The effect of Saline and Serp-1 treatment on gene expression in splenocytes in mice with PBMC infusion was assessed. Saline treated GCA^pos^ engrafted mice spleens had significantly increased gene expression for IL-1β, IL-17, and CD25 and reduced expression for fII on comparison with the GCA^neg^ implanted mice. IL-1β gene expression in GCA^pos^ engrafted mice was significantly reduced with Serp-1 tretament in PBMC transfused mice when compared to GCA^neg^ engrafted mice (Fig. 6A).

In combined data of GCA^pos^ and GCA^neg^ engrafted mice, Serp-1 significantly reduced gene expression for coagulation pathway proteases and receptors, specifically fІI, fX, tPA, the uPA receptor (uPAR), and protease activated receptor 2 (PAR2) in spleens from mice with PBMC infusion, when compared to saline treated controls (Fig. 6B). Other genes in the thrombotic and thrombolytic pathways were not altered (data not shown). Additionally, gene expression for immune cell markers, specifically CD3, CD4, Th1, Th17, Treg, IFN-γ, TNFα and IL-17α were significantly reduced with Serp-1 treatment (Fig. 6B). Serp-1 treatment produced a trend toward reduced expression for inflammatory cytokines, specifically IL-1β, IL-6, and IL-10, after PBMC transfusion, but these changes did not reach significance (data not shown).

## Discussion

With this study we have examined human TA biopsy xenografts from patients with suspected GCA after implant into the abdominal aorta of immunodeficient SCID mice. We detected significant increases in VIL size and mononuclear cell responses after 28 days follow up in GCA^pos^ grafts. Additionally, treatment with an anti-inflammatory serpin, Serp-1, significantly reduced VIL thickness and inflammatory cell infiltrates in GCA^pos^ grafts.

For these studies, a modified mouse model was developed using a full thickness TAI graft implanted into the anterior wall of mouse abdominal aorta. This provides a physiological interaction between the TAI and the circulating blood. Prior methods used subcutaneous implants without direct exposure to circulating blood [4]. GCA^pos^ TAI grafts developed greater inflammatory lesion growth and increased inflammatory cell responses when compared to GCA^neg^ grafts, indicating greater innate inflammation in positive biopsy sections.

As SCID mice have T- and B-cell deficiency, mice were also examined for responses to TAI engrafting after infusion with human PBMC in order to assess lymphocyte interactions with engrafted aorta. The presence of viable human PBMC was verified by colonization of human CD3^+^, MHC-1^+^, and mitochondria^+^ cells of the spleen and human T cells in the arterial TA grafts, associated with intimal thickening. Minimal human cell infiltrates were seen in adjacent non-grafted mouse aorta.

PBMC infusion increased the number of invading inflammatory cells in the engrafted artery medial layers. Compared to saline injected mice, VIL size in TAI with PBMC transfusion was larger, suggesting that PBMC infusions increased inflammation and arterial inflammatory lesion growth. The use of unmatched PBMC in the TAI model may also induce graft rejection and further investigation using matched PBMC infusion will be considered in future work. It should, however, be noted that prior research studies have reported no detectable graft-versus-host disease in mice with infusion of unmatched, allograft (xenograft) cells [20].

Glucocorticoids are highly effective in treating the constitutional symptoms of GCA, however, patients may require therapy for years to avoid recurrence, suggesting only partial responsiveness with ongoing risk for adverse side effects. Although steroids are believed to protect against sudden loss of vision [21], vascular lesions persist and disease can recur with steroid tapering. The sparing of Th1 arm of the mechanism of vascular lesion formation by glucocorticoid therapy [6] needs to be addressed for successful treatment of GCA.

In the present study, Serp-1 treatment was assessed as a new approach to targeting the thrombotic and thrombolytic pathways in circulating blood that may accelerate inflammatory cell activation in vascular lesions (VIL). Serp-1 has been previously extensively tested in animal models of vascular disease and arthritis with demonstrated significant prolonged reductions in plaque growth and inflammation after early short term treatment. Serp-1 treatment was also assessed in a recently completed Phase IIa clinical trial in patients with acute unstable coronary atherosclerosis, demonstrating significant reductions in markers for early myocardial damage after stent implant [19]. Assessment of Serp-1 in severe IVS is a logical next step and we have assessed Serp-1 as a potential treatment using TAI grafts from patients with suspected GCA and TA at risk for sudden blindness. We demonstrate here that Serp-1 reduced inflammatory cell infiltration and inflammatory lesion growth in xenopatch grafts from GCA^pos^ patients. In GCA^neg^ specimens, there was less reduction in VIL thickness and inflammation. Combined analysis of data from all GCA grafts without added PBMC, whether positive or negative on pathological analysis, detected reduced inflammatory lesion size and inflammation with Serp-1 treatment, and a greater reduction after PBMC infusion. These studies support potential for Serp-1 mediated attenuation of vascular inflammation in IVS.

TA biopsies from patients with GCA and IVS are reported to have ‘skip’ lesions with regions characteristically lacking evidence for arteritis. Thus biopsies reported as negative may have evidence of GCA in other areas. TAI grafts were examined in mice and some VIL growth may be secondary to activation of immune responses to the TA biopsy xenograft. While this cannot be entirely ruled out, the fact that the GCA^pos^ implants had greater VIL growth and greater responsiveness to serpin treatment when compared to GCA^neg^ sections suggests that, in part, the vasculitic transplants positive for GCA had a specific pathophysiology that is remedied by serpin treatment. Further analysis is necessary to validate this hypothesis.

In spleen cell isolates, Th1 and Th17 cell numbers and gene expression for IFN-γ (marker for Th1) and IL-17 (marker for Th17) were significantly reduced by Serp-1, in engrafted mice with PBMC transfusion. Of interest, splenocytes from mice with GCA^pos^ grafts also displayed increased Th and macrophage markers when compared to GCA^neg^ grafts. However, only CD11b^+^ monocytes were reduced with Serp-1 treatment, as observed in the histological sections of the TAI of both GCA^pos^ and GCA^neg^ groups. It is reported that T cells (Th1 and Th17) and macrophages are critical players in IVS inflammation in GCA [22]. Deng *et al* reported that glucocorticoid treatment more selectively suppresses Th17 responses, whereas Th1 responses were spared [6]. Thus to reset immune abnormalities in GCA, glucocorticoid treatment may have greater efficacy with the addition of other therapies [6], such as Serp-1, that in other models is reported to target macrophage, Th1 and Th17 cell responses [12].

Thrombosis is also associated with severe GCA lesions and ASA is used for treatment in IVS. Serp-1 inhibits thrombotic and thrombolytic serine proteases which activate inflammatory responses. Thrombotic and thrombolytic cascades are also activated, in turn, by activation of the innate immune response. We detected an array of significant changes in gene expression, with reduced FII, fX, and PAR-2 (a fX receptor), in the thrombotic pathway and tPA, plasminogen activator inhibitor-1 (PAI-1), and the uPA receptor (uPAR) in the thrombolytic pathway in Serp-1 treated mice. Serp-1 inhibition of aortic allograft plaque growth and inflammatory cell invasion has been previously noted to be blocked after implant of uPAR deficient mouse aortic allografts [10]. In GCA positive and negative engrafted mice, splenocytes also differed in gene expression for IL-1β, IL-17, and CD25, in saline treated mice. Serp-1 treatment reduced IL-1β after PBMC infusions.

Prior research has demonstrated that fX and tPA can promote cell proliferation, migration and differentiation [23]. Importantly, inhibition of fX reduces smooth muscle cell proliferation after balloon angioplasty [24]. Furthermore, in murine models, neointima formation is markedly reduced in uPA and PAR-2-deficient mice [25]. In a rat model of balloon-injured carotid artery, PAR-2 was up-regulated in neointima [26]. Therefore, Serp-1 may potentially reduce intimal plaque formation through inhibiting fXa and PAR2 in the thrombotic pathways or tPA, uPA and uPAR in the thrombolytic pathway. Additionally fX, tPA, PAR-2, and uPAR expression are enhanced in atherosclerotic lesions in human and in animal models, suggesting that coagulation pathway dependent cellular trafficking has the capability to drive inflammation in GCA [27]. When bound to PAR2, fX can induce secretion of pro-inflammatory cytokines, including IL-1 and IL-6 from fibroblasts [28], and mediates the expression of adhesion molecules on monocytes [29]. Fibrinolytic activators and inhibitors can alter inflammatory cell recruitment and migration. In particular, uPA and uPAR interact at the leading edge of invading mononuclear cells to activate matrix metalloproteases (MMPs) and allow leukocytes invasion in the arterial wall. The expression of uPAR on leukocytes is strongly associated with tissue invasion [30]. Since PAR2 and uPAR are located on the surface of monocyte, Serp-1 may directly inhibit immune cell invasion into arteries, in spleens when fX or uPA are bound to their receptors, or indirectly via inhibition of inflammation by suppression of cytokines such IL-1, IL-6, IL-10, IL-17, tumor necrosis factor alpha (TNFα) and interferon gamma (IFNγ) through altered gene expression. Although we can state that Serp-1 reduced both protease and inflammatory responses, specific mechanisms by which serpin treatment blocks (VIL) growth in TAI grafts will require further investigation.

In summary, a modified TAI, Aortic Window Xenograft vasculitis (IVS), model was established and altered tissue responses in GCA^pos^ TAI biopsies were detected. Significant changes in VIL growth and inflammatory cell invasion were detected with anti-inflammatory serpin treatment suggesting therapeutic potential for treatment of IVS.

## Supporting Information

S1 Table Immune cell types analyzed and corresponding fluorochrome-labeled antibodies utilized for flow cytometry. (DOCX)

S2 Table Primer Sequences Utilized in Quantitative RT-PCR Assays. (DOCX)
