# Supplementary material for: Serpin Treatment Suppresses Inflammatory Vascular Lesions in Temporal Artery Implants (TAI) from Patients with Giant Cell Arteritis
Source: PLoS One. 2015 Feb 6;10(2):e0115482. doi: 10.1371/journal.pone.0115482 (PMC4319900; doi:10.1371/journal.pone.0115482)
Supplement: S1 Table — (DOCX) [file pone.0115482.s001.docx]

**Table S1. Immune cell types analyzed and corresponding fluorochrome-labeled** **antibodies utilized for flow cytometry**

| **Cell type** | **Marker** |
| --- | --- |
| cytotoxic T cell | Anti-CD3-PerCP-Cy5.5; Anti-CD8-APC-eFluor780 |
| T helper cell | Anti-CD3-PerCP-Cy5.5; Anti-CD4-PE-Cy7 |
| Th1 cell | Anti-IFNγ-FITC |
| Th2 cell | Anti-IL4-PE |
| Th17 cell | Anti-IL17a-AF647 |
| Treg cell | Anti-FoxP3-eFluor450 |
| B cell | Anti-CD19-Cy7 |
| Hematopoietic stem cell  NK cell  monocyte  dendritic cell (Mature)  dendritic cell (Immature)  memory T cell | Anti-CD34-PerCP-Cy5.5  Anti-NK1.1-eFluor450  Anti-CD11b/c-APC-eFluor780  Anti-CD83-PE  Anti-CD206-FITC  Anti-CCR6-APC |
