# Supplementary material for: Serpin Treatment Suppresses Inflammatory Vascular Lesions in Temporal Artery Implants (TAI) from Patients with Giant Cell Arteritis
Source: PLoS One. 2015 Feb 6;10(2):e0115482. doi: 10.1371/journal.pone.0115482 (PMC4319900; doi:10.1371/journal.pone.0115482)
Supplement: S2 Table — (DOCX) [file pone.0115482.s002.docx]

**Table S2. Primer Sequences Utilized in Quantitative RT-PCR Assays.**

| **Gene name** | **Forward primer** | **Reverse primer** |
| --- | --- | --- |
| factor II | CCGAAAGGGCAACCTAGAGC | GGCCCAGAACACGTCTGTG |
| Factor X | GAGGGACACCTACGACTATG | GCCCAGTCTTTCTGAGGCA |
| uPAR | CAGAGCTTTCCACCGAATGG | GTCCCCGGCAGTTGATGAG |
| tPA | AACGCAGACAACTTACCAACA | GTTCGCTGCAACTTCGGA |
| PAR2 | TCCGGTCGTCTACATTATTGTGT | AGGGGGAACCAGATGACAGAG |
| IL-1 | AGCCCATCCTCTGTGACTCATG | GCTGATGTACCAGTTGGGGAAC |
| IL-6 | TAGTCCTTCCTACCCCAATTTCC | TTGGTCCTTAGCCACTCCTTC |
| IL-10 | GCTCTTACTGACTGGCATGAG | CGCAGCTCTAGGAGCATGTG |
| IL17 | TCAGCGTGTCCAAACACTGAG | GACTTTGAGGTTGACCTTCACAT |
| TNFa | CCCTCACACTCAGATCATCTTCT | GCTACGACGTGGGCTACAG |
| IFNγ | ATGAACGCTACACACTGCATC | CCATCCTTTTGCCAGTTCCTC |
| CD3 | GTGGAACACTTTCTGGGGCATCCTG | TGTTCTCGGCATCGTCCTGGCA |
| CD4 | ACCATGTGCCGAGCCATCTCTCTT | CCAGCACCAGCGTCTTCCCTTG |
| GAPDH | AGGTCGGTGTGAACGGATTTG | TGTAGACCATGTAGTTGAGGTCA |
